# Zinc oxide calcium silicate composite attenuates acute tramadol toxicity in mice

**DOI:** 10.1186/s40360-023-00647-0

**Published:** 2023-02-09

**Authors:** Shaimaa A. ElShebiney, Rania Elgohary, Sayed H. Kenawy, Gehan T. El-Bassyouni, Esmat M. A. Hamzawy

**Affiliations:** 1 Narcotics, Ergogenics, and Poisons Department, National Research Centre, 33-El-Buhouth St., 12622 Dokki, Giza, Egypt; 2 Refractories, Ceramics and Building Materials Department, National Research Centre, 33 El Buhouth St, Dokki, Giza, 12622 Egypt; 3 Glass Research Department, National Research Centre, 33 El Buhouth St, Dokki, Giza, 12622 Egypt

**Keywords:** Adsorption, Antidote, Silicate phases, Nano-microstructure, Toxicity management, Wollastonite

## Abstract

**Background:**

Seizures are considered to be the most common symptom encountered in emergency- rushed tramadol-poisoned patients; accounting for 8% of the drug-induced seizure cases. Although, diazepam clears these seizures, the risk of central respiratory depression cannot be overlooked. Henceforth, three adsorbing composites were examined in a tramadol acute intoxication mouse model.

**Methods:**

Calcium Silicate (Wollastonite) either non-doped or wet doped with iron oxide (3%Fe_2_O_3_) or zinc oxide (30% ZnO) were prepared. The composites’ adsorption capacity for tramadol was determined in vitro. Tramadol intoxication was induced in Swiss albino mice by a parenteral dose of 120 mg/kg. Proposed treatments were administered within 1 min at 5 increasing doses, i.p. The next 30 min, seizures were monitored as an intoxication symptom. Plasma tramadol concentration was recorded after two hours of administration.

**Results:**

The 3% Fe_2_O_3_-containing composite (CSFe3), was found to be composed of mainly wollastonite with very little alpha–hematite. On the other hand, hardystonite and wellimite were developed in the 30%ZnO-containing composite (CSZn3). Micro-round and irregular nano-sized microstructures were established (The particle size of CS was 56 nm, CSFe3 was 49 nm, and CSZn3 was 42 nm). The CSZn3 adsorption capacity reached 1497 mg of tramadol for each gram. Tramadol concentration was reduced in plasma and seizures were inhibited after its administration to mice at three doses.

**Conclusion:**

The calcium silicate composite doped with ZnO presented a good resolution of tramadol-induced seizures accompanied by detoxification of blood, indicating its potential for application in such cases. Further studies are required.

## Background

Tramadol abuse is an expanding health and economic problem, which is prevalent not only in the Egyptian population [1], but also worldwide [2]. In October 2012, the alarming epidemiological data reviews and surveillance research caused the Egyptian government to list the drug under national control [3, 4]. Among the young population in Egypt, tramadol is the first drug of choice for abuse owing to the fact that it resembles more than 40% of the abused drugs [1, 5]. Tramadol acquires additional rewarding effects such as enhancing men’s sexual activity, which reinforces its use [6, 7].

Controversially, the most concerning overdose symptom with a relatively high incidence is tramadol-encountered seizures [8–11]. They account for 8% of drug-induced seizure reported cases [12]. Encountered seizures are described by several reports after the chronic use of tramadol for pain management, recreational purposes or after a single administration of more than 500 mg dose [13–16].

In Egypt, the incidence of seizures as a tramadol intoxication symptom represents 35% of the clinical toxicology centers admitted patients. Besides, about 7% of seizure-complaining patients at epilepsy clinics were attributed to tramadol repeated administration [17]. The management of tramadol-poisoned patients with naloxone results in different actions depending on the individual responses. Some reports show decreased seizure episodes, while others report preserved or increased incidence [18, 19]. Despite of its CNS depressant risk, diazepam is the drug of choice for seizures management [20].

Generally, detoxifying actions involve multiple mechanisms including: limiting absorption, poison sequestration, inhibiting the metabolism of a toxic metabolite, promoting distribution from tissues, poison displacement/competition for the receptor or counteracting the toxic effect and enhancing detoxification [21]. To manage tramadol intoxication, treatment should not only rely on opioid receptor modulation, symptomatic treatment for toxicity associated incidents is of high value as well [22].

Wollastonite is a characteristic inorganic compound composed of calcium silicate, which can be derived from naturally occurring limestone and diatomaceous earth or produced chemically from the reaction of calcium oxide and silica [23]. Calcium silicate is added pharmaceutically as an inactive ingredient for industrial purposes [24] due to its specific physical characteristics such as uniform structure, and large pore volume and surface area. It is an excellent material for drug delivery systems since it has a large surface area for drug adsorption having no cytotoxic effects [25–27].

It is widely used as a biomedical material for several purposes, such as bone tissue engineering and dental caries [28, 29]. Doping wollastonite with trace element oxides, such as Zn is a common practice that benefits from their bioavailability and bioactivity. These oxides can be impregnated into the calcium silicate material by several preparation techniques. The wet method provides excellent chemical and structural homogeneity, it derives metastable structures at low reaction temperatures. It is considered a green chemistry method [30, 31].

Consequently, the present study applied the doping technique to replace a small percentage of calcium ions in the wollastonite composite with other elements, such as zinc and iron, to achieve tramadol anti-toxicity action in an animal model of tramadol intoxication. It also compared the produced effect with diazepam, the common management aid.

## Methods

### Preparation of wollastonite

Using the wet precipitation method, wollastonite [CaSiO_3_, (CS)] was prepared from the starting materials calcium carbonate [CaCO_3_, El-Gomhouria Company for Trading Chemicals and Medical Appliances, Cairo, Egypt], and silica gel (amorphous SiO_2_; Fluka Chemie GmbH, Sigma-Aldrich, Buchs, Switzerland). Upon mixing the mentioned contents in the specified ratios (Table 1), the resultant solution was dehydrated in a dryer for one day at 100℃, thermally preserved, before being milled in a ball mill (Model Retsch GmbH 5657 Germany, Type S1, Volt 220/50 Hz) to get hold of the powder.

**Table 1:** Nominal chemical composition of composites

Composite symbol	ZnO ratio %	Fe_2_O_3_ ratio %	Constituents			
			CaCO_3_	SiO_2_	^a^Zn(CH_3_CO_2_)_2_	^2^Fe(NO_3_)_3_
CS	0	0	84.42	51.72	0	0
^1^CSFe3	0	3%	84.42	51.72	0	4.59
^2^CSZn3	30%	0	55.51	48.53	53.13	0

^1^ incorporation of 3% Fe_2_O_3_, ^2^ Incorporation of 30% ZnO in calcium silicate (CS) composition

### Doping of wollastonite with iron (CSFe3)

Firstly, Calcium nitrate Ca(NO_3_)_2_ solution was attained by liquifying CaCO_3_ in an appropriate amount of concentrated nitric acid. Silica gel and iron nitrate [Fe(NO_3_)_3_, BDH, England] were consequently involved individually as ancestors for SiO_2_ and Fe^3+^. 3% Fe_2_O_3_ in 100 g of the wollastonite slurry was formulated. The resulting gel was left to age with magnetic stirring to guarantee complete homogeneity and mixing. Afterwards, the formed gel was dried at 100℃, thermally treated up to 1000℃/2 h, crushed into fine powder, and sieved by a standard sieve of size 63 μm.

### Doping of wollastonite with zinc (CSZn3)

During the course of the wet precipitation, CS was subjected to fractional substitution of CaO by ZnO (in CS:ZnO ratios of 7:3). The starting materials were CaCO_3_, zinc acetate [Zn (CH_3_CO_2_)_2_] presented as the dihydrate Zn(CH_3_CO_2_)_2_⋅2H_2_O (Qualikems Fine Chemicals Pvt. Ltd., New Delhi, India) and silica gel. Ca(NO_3_)_2_ solution was attained as described before and added to aqueous solution of Zinc acetate (30%) and Silica gel, the resultant solution was dried in a dryer for one day at 100℃, thermally treated, then pulverized in a ball mill to obtain powder with particle size 0.083 mm.

### Characterization techniques

The phase identification of the composites was performed using the X-ray diffraction analysis (XRD, Advanced Bruker D8 Diffractometer-Philips, PW1390, Germany). High resolution scanning electron microscopy (HR-SEM) was used to explain the microstructure of the pre-treated samples (HR-SEM, Philips Model-FEG Quanta 250 with field emission gun FEI, Netherlands).

### Adsorption capacity

Tramadol stock solution of 50 mg/ml was prepared. 100 mg of powdered composites were weighed and mixed with 0.75 ml or 1.5 ml of stock tramadol HCL solution and completed to 10 ml by Ringer’s simulated body fluid in triplicates. The mixture was sonicated at 37 °C for 15 min and kept still for other 15 min. then, mixture was centrifuged at 4000 rpm for 10 min and the supernatant was brought to pH 10 by drops of 10N NaOH and extracted and analyzed using HPLC method as described in Sect. 2.7. A standard solution of both concentrations was prepared in duplicates and proceeded in same experimental procedures and analyzed.

### Animals and drugs

Male inbred Swiss mice of NRC breeding colony (5–6 weeks, 25–30 g) were used. Animals were housed in plastic cages (4 per cage), maintained in controlled laboratory conditions (23–26 °C, 30–50% relative humidity, 12 h light/dark cycle, lights on 6:00 a.m.) and kept on standard diet and tap water ad libitum. Animals were acclimatized for a week to the experimental room, where behavioral experiments were carried out. All experimental procedures were conducted in accordance with the National Institutes of Health guide for the care and use of Laboratory animals (NIH Publications No. 8023, revised 1978) and were reviewed and approved by the Institutional Animal Care and Use Committee of NRC. Reporting of experimental data was in accordance with the ARRIVE guidelines.

Tramadol HCl was generously provided from ADWIA, Egypt. Tramadol was dissolved in sterile water (50 mg/ml). Different powdered composites were dispersed by tween 80 in sterile water to make 10 mg/ml solution. Diazepam (2 mg/ml vial, Valium, Egypt) was diluted in sterile water (1 mg/ml). Sodium Pentobarbital (50 mg/ml, Egypt) was applied for anesthesia purposes.

### Experimental protocol

Group size was calculated using G power software with effect size of *f* = 0.23 and power of 0.95 and determined to be 8 mice per treatment [32]. Test treatments included three wollastonite composites (CS, CSFe3, and CSZn3), diazepam and sterile water. A total of 136 mice were subjected to seizures induced by a single i.p. injection of tramadol HCl solution (120 mg/kg, i.e. 40% of LD50, Bameri et al., 2018). Animals that showed lasting seizure episode for more than 1 min were euthanized. Within 1 min, one group (n = 8) kept as ***tramadol*** control received sterile water (1 ml/kg, i.p.) and another one (n = 8) received ***diazepam***, (1 mg/kg, i.p., Herrera-Calderon et al., 2018). Other groups were assigned for five escalating doses (20, 40, 80, 160, 320 mg/kg, i.p.) from ***CS, CSFe3*** and ***CSZn3*** composites (*n* = 8 for each dose).

**Fig. 1:**
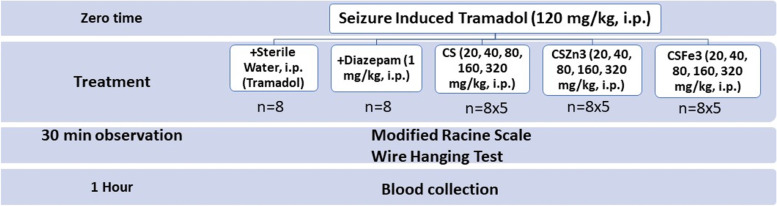
Experimental protocol

Seizure scoring was performed right after injection by a blind observer for 30 min (between 10 and 11 a.m.). After one hour of tramadol administration, pentobarbital (50 mg/kg, i.p.) was administered, blood was collected in EDTA tubes via retro-orbital plexus, plasma was separated by centrifugation at 3000 rpm for 10 min, and stored at -20℃ (Fig. 1).

### Assessment of tramadol-induced seizures and wire hanging test

After drug administration, seizures were scored for 30 min by a blind observer using scoring scale of 0 to 5, where 0:no seizures, 1: twitches, 2: tremors and twitches, 3: general stretches and tremors, 4: one sided tonic convulsions, 5: tonic–clonic convulsions [33].

Subsequently, mice were hung on a stainless-steel wire 30 cm above the surface without support. The time elapsed to fall was recorded till 30 s at most [34].

### Estimation of tramadol concentration

Plasma levels of tramadol were measured using HPLC analysis. Plasma samples were liquid–liquid extracted by ethyl acetate:hexane (1:4, *v/v*) in three replicates. The extract was evaporated and re-dissolved in 1 ml of methanol (HPLC grade) for HPLC analysis. HPLC was performed using Dionex Ultra 3000 PDA, C-18 column (Zorbax, 15 cm × 25 µm × 4.6 µm). Mobile phase was freshly prepared, 0.1% Trifluoroacetic acid: methanol: acetonitrile (30:25:45) and set to 1 ml/min. PDA detection set at 218 nm. Standard curve was constructed at the beginning of analysis and data was automatically extrapolated.

### Statistical analysis

Data was statistically analyzed using one-way ANOVA followed by Tukey's multiple range comparison as post hoc test. Statistical analysis for mortality data was done using chi-squared test. Seizure scores were performed by Kruskal–Wallis non-parametric one-way analysis of variance (ANOVA), score data are expressed as median ± Interquartile Range (IQR). Values were considered significant when *p* < 0.05. Spearman’s rank order correlation coefficient was calculated for plasma tramadol level and seizure score. Graphpad Prism (v.10) was used for performing analysis and graph construction.

## Results and Discussion

### Characterization of developed treatments

**Fig. 2:**
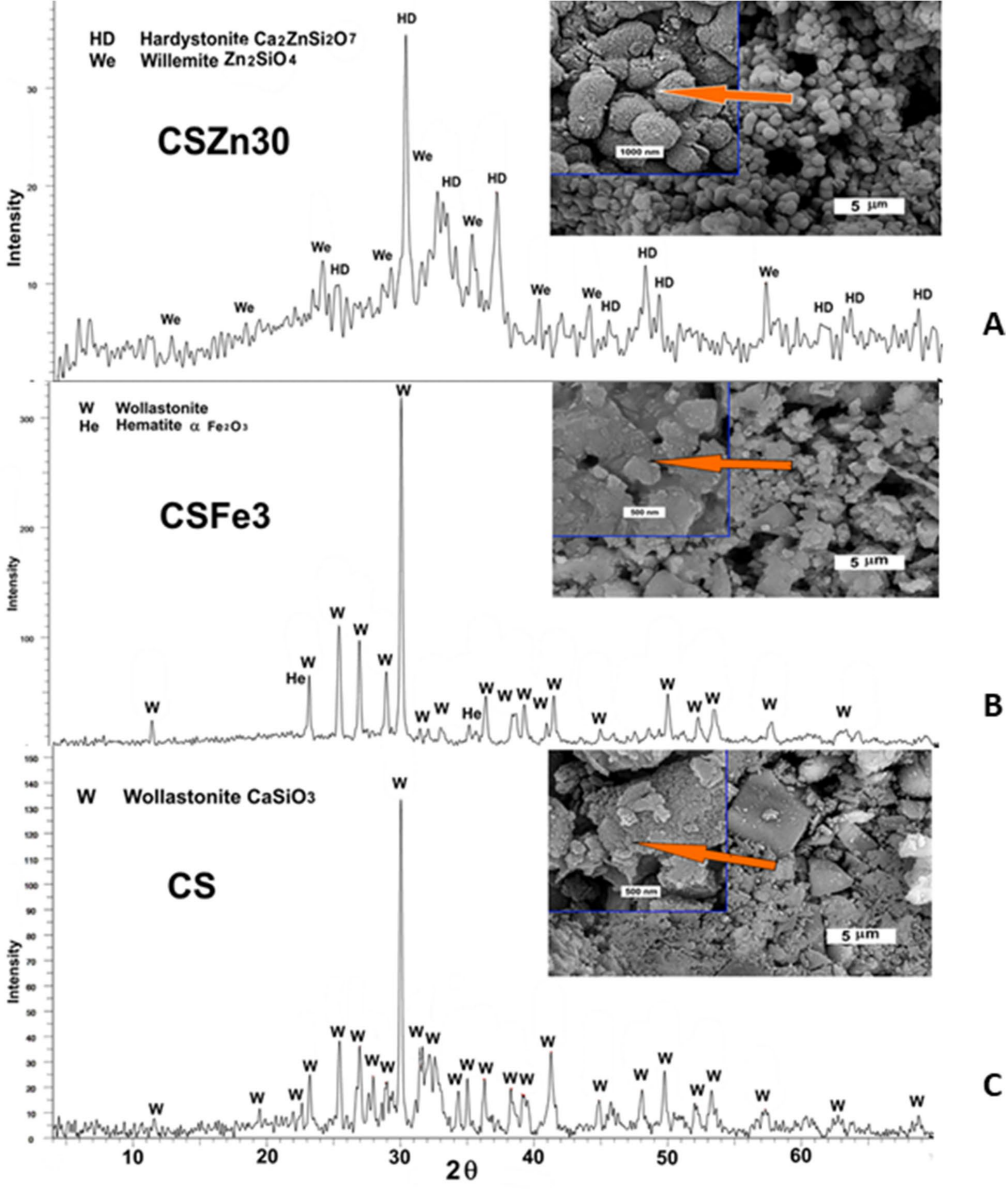
XRD and HR-SEM examination of the developed composites. **A** 30%ZnO-doped wollastonite showing crystallization of hardystonite and willemite, SEM photo outlines the rounded shape nanoparticles (arrow), **B** 3%Fe_2_O_3_-doped wollastonite showing crystals of alphahematite in addition to its irregular shape in SEM photo (arrow), **C** the undoped wollastonite showing its purity and irregular shaped nanoparticles (arrow)

Although, wollastonite (CaSiO_3_, ICDD, 01–084-0655) was the main developed phase in the parent CS and CSFe3 composite, traces of alpha-hematite (α-Fe_2_O_3_, ICDD-73–0603) were detected in CSFe3 composite. On the other hand, hardystonite (Ca_2_ZnSi_2_O_7_ -JCPDS 01–075-0916) and willemite (α-Zn_2_SiO_4_, ICDD Card No. 00–037-1485) were crystallized in CSZn3 [26, 29]. The developed crystalline phases and the microstructures in the composites are shown in Fig. 2. In case of CS and CSFe3, the microstructure showed nanoparticles of the former phases accumulated in irregular outline particles, or in the form of rounded clusters as in case of CSZn3 (Fig. 2). The particle size of CS, CSFe3 and CSZn3 evaluated from the maximum peak width of the X-ray pattern, in support of the Scherrer formula, were 56, 49 and 42 nm, respectively. This outcome demonstrated that the samples under investigations were designed as nano-crystalline materials.

### In vitro study of adsorption capacity of prepared composites

The adsorption capacity of the different powders for tramadol HCl in Ringer’s solution was shown to be 1125.15 ± 72.3 mg tramadol/g CS composite, 1497.4 ± 0.56 mg tramadol/g CSZn3 composite and 358.1 ± 30.4 mg tramadol/g CSFe3 composite.

### In vivo effect of different treatments on tramadol induced seizures

Tramadol (120 mg/kg) induced seizures of a severity score median (4.0) and caused more than 50% mortalities in mice. Standard treatment by diazepam (1 mg/kg) caused great reduction of the seizure score compared to positive control group (median 0.14) but high mortality rate was recorded (41.6%) which is not significant from that of the positive control group.

Administration of CS composite showed median seizure score of 2.3 and 3.3 at 160 and 320 mg/kg doses, whereas the lower doses 20, 40 and 80 mg/kg scored 4.1, 3.2, and 3.6, respectively. The applied CSZn3 treatment had a dose dependent decline of median seizure score after the administration of 20 mg/kg (2.4), 40 mg/kg (2.5), 80 mg/kg (1.3), 160 mg/kg (0.8), and 320 mg/kg scoring 2.2, an effect that was significant at the doses 80, 160 and 320 mg/kg. On the other hand, only was the 80 mg/kg of CSFe3 treated mice score significant from tramadol positive group.

**Fig. 3:**
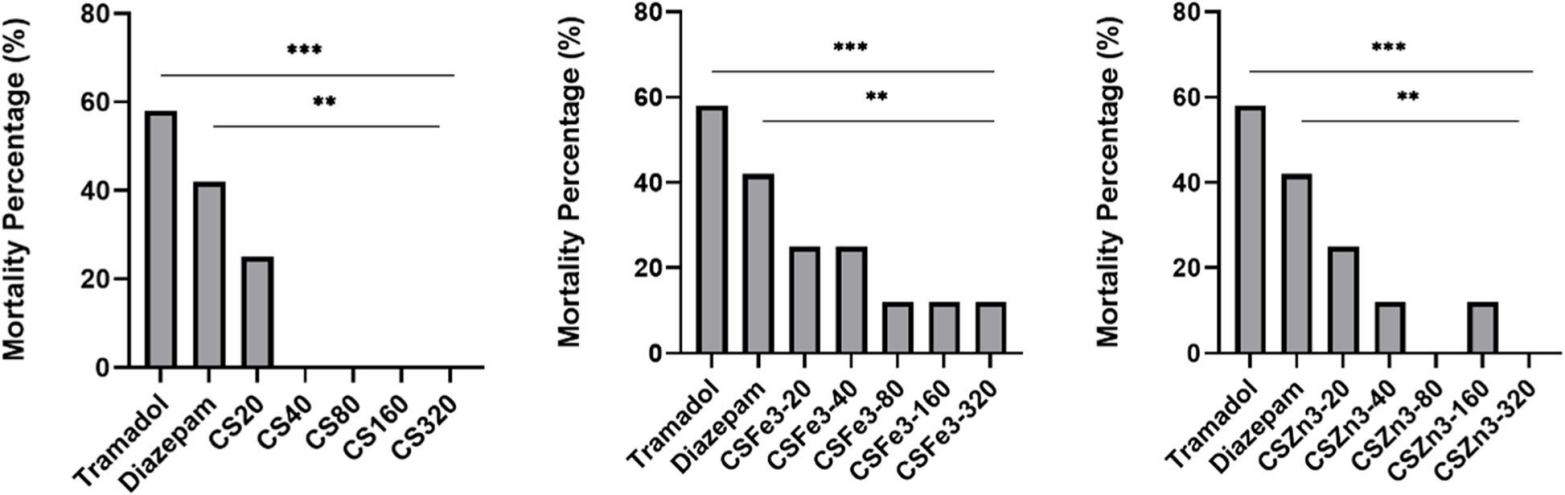
Mortality rate after different treatments of tramadol intoxicated mice. Data represents mortality percentage statistically analyzed using chi-squared test.** For comparison against diazepam, *** for comparison against tramadol

**Fig. 4:**
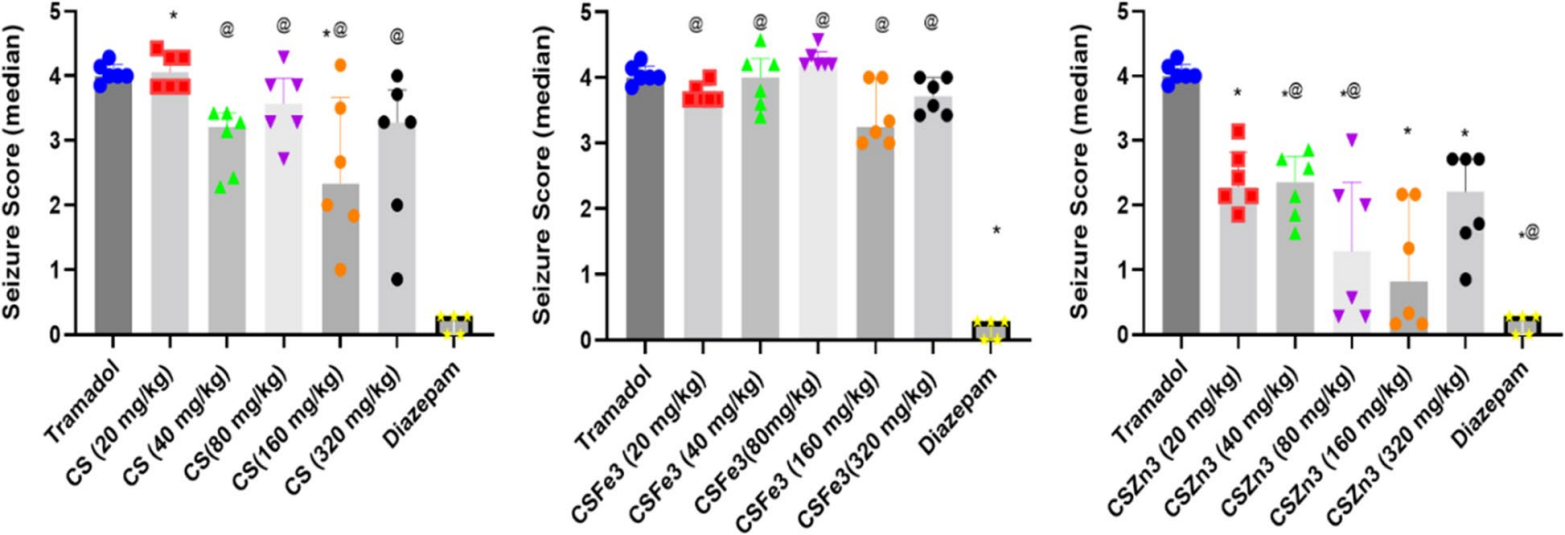
Seizure severity of tramadol intoxicated mice after management with developed treatment at different doses of CS, CSZn3 or CSFe3. Data represents median and interquartile range (*n* = 7) across 30 min of observation, *p < 0.05 versus tramadol, ^@^ For comparison against diazepam using the post-test Kruskal–Wallis with non-parametric one-way ANOVA

Though developed composites improved mortality percentage in comparison to both tramadol- (50%) and diazepam- (42%) treated animals (*p* < *0.05*). Mortality of animals was abolished by CS treatment, as only 25% of the animals died after the 20 mg/kg treatment dose, while no deaths occurred after any of the used CS doses. Moreover, CSZn3 reduced mortality to 12.5% (p < 0.05) at 40 and 160 mg/kg doses, while the other doses did not show any mortality. CSFe3 treatment at doses 20 and 40 mg/kg reduced mortality to about 25%. On the hand, the 80, 160 and 320 mg/kg doses of CS caused only 12.5% mortality. Data is displayed in Figs. 3 and 4.

### Effect of tramadol intoxication and treatment on wire hanging time

Tramadol (120 mg/kg) intoxication caused a deterioration of the muscle grip strength which was reflected on the falling time from a hanging wire (2 ± 0.58 s). Diazepam treatment caused mild enhancement that was around 4 ± 3.5 s. (Normal hanging time was 28 ± 1.9 s, data not shown,).

Applied treatments showed diverse effects; the CS-treated animals recorded 14.6 ± 4.6, 16 ± 4.2, 17.14 ± 3.35, 20.3 ± 3.9 and 16.3 ± 3.6 s at the doses of 20, 40, 80, 160 and 320 mg/kg. The falling time of mice treated with different doses of CS was significant from tramadol and diazepam treated mice (*p* < 0.05). Tested doses were not statistically different.

The CSFe3 showed enhanced grip strength compared to tramadol (*p* < 0.05) intoxicated mice that reached 4.3 ± 3.1, 10 ± 3.4, 4.2 ± 0.52, 19.8 ± 4.2, and 17.3 ± 3.9 s after its administration at the doses of 20, 40, 80, 160 and 320 mg/kg, respectively. The 160 and 320 mg/kg doses effect was significant from the effect of 20 and 80 mg/kg doses and diazepam-treated mice. In addition, management of tramadol intoxicated animals by CSZn3 achieved better hanging time compared to tramadol-treated mice (*p* < 0.05) at the doses of 20, 40, 80, 160 and 320 mg/kg (11.5 ± 3.4, 8.6 ± 3.5, 23.6 ± 4.1, and 27.3 ± 1.8 and 14.8 ± 3.2 s, respectively). The effect was markedly enhanced than diazepam at the doses of 80, 160 and 320 mg/kg (*p* < 0.05). It is noteworthy that the recorded time for CSZn3 treated animals at doses 80 and 160 mg/kg was comparable to the normal grip time (Table 2).

**Table 2:** Wire hanging time (seconds) of tramadol-intoxicated mice and the effect of different treatments

Treatment	20 mg/kg	40 mg/kg	80 mg/kg	160 mg/kg	320 mg/kg	Tramadol	Diazepam
**CS**	14.6 ± 4.6*^@^	16 ± 4.2*^@^	17.14 ± 3.35*^@^	20.3 ± 3.9*^@^	**16.3 ± 3.6**	**2 ± 0.58**	**4 ± 3.5**
**CSFe3**	4.3 ± 3.1*	10 ± 3.4*	4.2 ± 0.52*	**19.8 ± 4.2**^ac^*^@^	**17.3 ± 3.9**^ac^*^@^	**2 ± 0.58**	**4 ± 3.5**
**CSZn3**	11.5 ± 3.4*	8.6 ± 3.5*	**23.6 ± 4.1**^b^*^@^	27.3 ± 1.8*^ab c@^	14.8 ± 3.2*^@^	**2 ± 0.58**	**4 ± 3.5**

Normal wire hanging time (seconds) was recorded to 28 ± 1.9 s (data not shown). Data are mean ± SEM, *n* = 7. Significance at *p* < 0.05 using one-way ANOVA followed by Tukey's multiple range test. ^a^ For comparison against 20 mg/kg, ^b^ For comparison against 40 mg/kg, ^c^ For comparison against 80 mg/kg, ^d^ For comparison against 160 mg/kg, * For comparison against tramadol, ^@^ For comparison against diazepam

*CS* wollastonite-treated mice, *CSFe3* wollastonite doped with 3% iron oxide, *CSZn3* wollastonite doped with 30% zinc oxide

### Plasma tramadol concentration

Plasma concentration after one hour of intraperitoneal injection of 120 mg/kg tramadol HCl reached 2679.85 ± 30.84 ng/ml**.** The plasma level after treatment with diazepam was not significant from untreated mice (2166.23 ± 58.12 ng/ml). Treatment of tramadol intoxicated mice with CS at the doses of 80, 160 and 320 mg/kg produced significant reduction of plasma tramadol concentration (1342.22 ± 10.64, 692.98 ± 10.78, and 836.04 ± 26.81 ng/ml, *p* < 0.05) in comparison to untreated mice and diazepam-treated mice. However, the CS lower doses 20 and 40 mg/kg did not change the plasma tramadol concentration (2868.65 ± 63.25 and 2789.27 ± 58.91 ng/ml). Administration of CSFe3 (160 mg/kg) caused tramadol level to fall to 1695.2 ± 31.8 ng/ml (p < 0.05), while the other CSFe3 treatment doses resulted in insignificant plasma concentration from untreated intoxicated mice (2747.85 ± 32.32, 2076.59 ± 27.78, 2361.67 ± 675.34, and 2084.42 ± 402.79 ng/ml).

On the other hand, the tramadol plasma level in CSZn3 treated mice was significantly lower than tramadol and diazepam-treated mice at all the administered doses, *p* < 0.05. However, the 320 mg/kg dose of CSZn3 showed higher plasma tramadol concentration compared to all the other doses (*p* < 0.05) (Table 3).

**Table 3:** Plasma tramadol concentration after different treatments

**Treatment**	**20 mg/kg**	**40 mg/kg**	**80 mg/kg**	**160 mg/kg**	**320 mg/kg**	**Tramadol**	**Diazepam**
**CS**	2868.65 ± 63.25^@^	2789.27 ± 58.91^@^	1342.22 ± 10.64^ab^*^@^	692.98 ± 10.78^abc^*	836.04 ± 26.81^abc*@^	2679.85 ± 30.84	2166.23 ± 58.12
**CSFe3**	2747.85 ± 32.32	2076.59 ± 27.78	2361.67 ± 675.34	1265.63 ± 187.54^*@^	2084.42 ± 402.79	2679.85 ± 30.84	2166.23 ± 58.12
**CSZn3**	899.61 ± 6.89*^@^	881 ± 1.34*^@^	694.83 ± 22.27^ab^*^@^	564.78 ± 23.88^ab^*^@^	1088.26 ± 79.6^abcd^*^@^	2679.85 ± 30.84	2166.23 ± 58.12

Plasma samples were extracted 1 h after tramadol administration and stored at -20℃ till analysis. Data represents average of 4 mice ± SEM. Significance at p < 0.05 using one-way ANOVA followed by Tukey's multiple range test. a For comparison against 20 mg/kg, b For comparison against 40 mg/kg, c For comparison against 80 mg/kg, d For comparison against 160 mg/kg, * For comparison against tramadol, @ For comparison against diazepam

*CS* wollastonite-treated mice, *CSFe3* wollastonite doped with 3% iron oxide, *CSZn3* wollastonite doped with 30% zinc oxide

Plasma tramadol concentration was positively correlated with the seizure score (*ρ* = 1, 0.9, 0.7 for the CS, CSFe3, and CSZn3, respectively) except in case of diazepam (*ρ* = 0.43) where seizure score was reduced but plasma level was not significant from the untreated mice. The CS (80, 160 and 320 mg/kg) and CSZn3 (20, 40, 80, and 160 mg/kg) showed the most preferable plasma level which is significantly lower than the untreated tramadol group plasma level.

## Discussion

Tramadol overdose is responsible for consciousness impairment, self-limiting generalized tonic–clonic seizures and possible induced trauma, agitation, respiratory depression, and serotonin syndrome [13, 35, 36].

The management of tramadol-poisoned patients with naloxone remains controversial. The benzodiazepine/tramadol combination consistently resulted in worsened CNS depression, in both animals and humans making it a risky choice [37, 38]. The current study succeeded in inducing seizures in more than 80% of animals after tramadol intoxication, providing a valid model for studying tramadol intoxication in mice. The seizure severity was rated to 4 and was resolved by diazepam injection.

Previously, [39] used single injection of tramadol (75 mg/kg, i.p.) to SD rats to induce seizures. [40] induced seizures in mice by injecting intravenously delivered tramadol solution that its dose reached the same dose range used in our study. Our findings were consistent with their findings, where tramadol-associated seizures were diminished after diazepam administration. However, CNS depression which is related to the high mortality rate was observed.

The seizure activity of tramadol can be related to opioid receptors’ overactivation. Since opioid delta receptor entails proconvulsant effects, high doses of tramadol can produce seizures by activating this receptor as well [41, 42]. Furthermore, an opioid-dependent GABA inhibitory pathway activation can be linked to tramadol-associated seizures [43]. The active metabolite O-desmethyltramadol is of high affinity to Mu and delta-opioid receptors and contributes to serotonergic effect as well [42, 44]. It was reported to induce seizures in mice though less potent than tramadol [45]. Seizures associated with tramadol overdose usually do not respond to naloxone but are relieved with benzodiazepines. Naloxone can be used for the treatment of post-seizure complaints [46]. A combination of diazepam/naloxone is reported as an efficient antidote to reverse tramadol-induced CNS toxicity [39].

At the highest dose level tested, CS reduced the plasma tramadol level and seizure score. The current study revealed that CSZn3 was the best remedy for induced seizures and its effect correlated with the decrease in plasma tramadol concentration. On the other hand, CSFe3 did not reduce the seizure score or the plasma tramadol level except at the dose of 160 mg/kg which caused a significant reduction in the plasma tramadol level reflected on the seizure score.

The bioactive material (CSZn3) saved at least 20–30% of animals from mortality encountered after tramadol poisoning and even could have saved 100% of the animals at certain dose levels. Our results showed a dose-dependent amelioration of seizure score reflected on plasma tramadol concentration that was maximal at a dose of 160 mg/kg.

The essential trace element (Zn) was suggested to modulate GABA receptors [47] or induce synaptic membrane depolarization [48], which directed the search for its role in the case of tramadol intoxication.

Previous studies proved a role for Zn ion in opioid receptor mediated physiological actions such as the study of [49] who observed that Zn chelators increased opioid withdrawal manifestations. Moreover, zinc oxide nanoparticles enhanced the analgesic effects produced by tramadol or morphine acute administration providing additional evidence of opioid receptor modulation [50]. Furthermore, Zn acts as a blood brain barrier stabilizer and maintains its integrity in pathological conditions [51]. The role of Zn in tramadol overdose-induced seizures can be crucial; however, it needs further studies. CSZn3 effect declined at the highest dose (320 mg/kg), which may be attributed to the neurotoxic effect of Zn at high doses, where excess Zn acts biophysically on NMDA receptors regulating glutamate release thus promoting excitotoxicity [52]. Besides, excessive extracellular zinc concentrations are responsible for increased oxidative damage [53].

Interestingly, mice were void of mortality after the administration of a dose of 160 mg/kg of either CSZn3 or CS, indicating that the composite related antidote action does not depend solely on the resolution of seizures; it also counteracts tramadol general depressant effects and lowers its circulating level.

Iron micronutrient is critical for neuronal health. Its deficiency was linked to increased seizure susceptibility [54]. Nevertheless, high iron concentration is linked to promoted oxidative damage and induces inflammatory response [54]. Also, iron deposition, as a result of high concentrations, is found in epileptic brain areas [55]. This can explain the current observation of unaffected seizure score of tramadol-intoxicated mice treated with CSFe3 despite the lowered tramadol plasma level.

The calcium silicate nano-biomaterial is a good candidate for drug delivery and increasing the efficacy of loaded drugs for its enhanced chemical and physical characteristics [56]. In addition, it can provide additional value to the desired therapeutic application. This study showed its ability to decrease tramadol plasma concentration though its specific action has not been investigated yet.

## Conclusion

It can be concluded from the presented data that the calcium silicate base was a good effective biomaterial to be applied in the cases of tramadol intoxication. Doping with zinc boosts the anti-toxic activity and represents a promising tool for future research in this perspective. The current study provides a prospective for the management of tramadol toxicity in hospitalized patients through the parenteral injection of a nano-developed safe biomaterial. These safe biomaterials containing trace elements, assist to balance the affected patients and help the medical staff to stabilize the clinical symptoms of seizures.

A limitation of the study is the need for mechanistic investigation and monitoring of the tramadol metabolite level for providing an overall vision. Indeed, further work is substantial to adopt future potential applicability in the clinical settings.

## Data Availability

The datasets used and/or analysed during the current study are available from the corresponding author on reasonable request.
